# SNS-CF: Siamese Network with Spatially Semantic Correlation Features for Object Tracking

**DOI:** 10.3390/s20174881

**Published:** 2020-08-28

**Authors:** Thierry Ntwari, Hasil Park, Joongchol Shin, Joonki Paik

**Affiliations:** Graduate School of Advanced Imaging Science, Multimedia and Film, Chung-Ang University, Seoul 06974, Korea; jonthierry@ipis.cau.ac.kr (T.N.); hspark@ipis.cau.ac.kr (H.P.); jcshin@ipis.cau.ac.kr (J.S.)

**Keywords:** object tracking, siamese network, correlation filter, spatially semantic correlation features

## Abstract

Recent advances in object tracking based on deep Siamese networks shifted the attention away from correlation filters. However, the Siamese network alone does not have as high accuracy as state-of-the-art correlation filter-based trackers, whereas correlation filter-based trackers alone have a frame update problem. In this paper, we present a Siamese network with spatially semantic correlation features (SNS-CF) for accurate, robust object tracking. To deal with various types of features spread in many regions of the input image frame, the proposed SNS-CF consists of—(1) a Siamese feature extractor, (2) a spatially semantic feature extractor, and (3) an adaptive correlation filter. To the best of authors knowledge, the proposed SNS-CF is the first attempt to fuse the Siamese network and the correlation filter to provide high frame rate, real-time visual tracking with a favorable tracking performance to the state-of-the-art methods in multiple benchmarks.

## 1. Introduction

Visual object tracking aims at estimating the position of an arbitrary target in a video sequence by establishing a correspondence between similar pixels of different frames [1,2,3]. It finds a wide range of usage in intelligent video analysis applications such as automatic visual surveillance, autonomous driving, augmented reality, and action recognition tasks, to name a few.

Despite the tremendous progress of visual tracking over the past few years, we still face the rise of numerous challenges including fast motion, illumination variation, occlusion, background clutter, intraclass variations, and so forth.

To alleviate the above challenges, we will learn better and more robust features that improve visual object tracking algorithms [4]. We adopted the same idea to deep learning algorithms using the most important features in the network. Another remedy for the above challenges is that object tracking changed its gears to an alternative approach in which, a Deep CNN is trained to address a more general similarity learning (Siamese learning) problem in an initial offline phase, and then this function is simply evaluated online during tracking, as explained in Bertinetto and Luca et al. [5].

Thanks to the findings that the straightforward replacement of shallow backbone with deeper and wider networks does not bring much improvement to Siamese network, the notorious accuracy gap to Siamese network counterparts is remarkable as described in References [1,2,6] but still, Reference [3,7] proved that spatially semantic correlation features are necessary to boost even further the accuracy gap.

**Figure 1:**
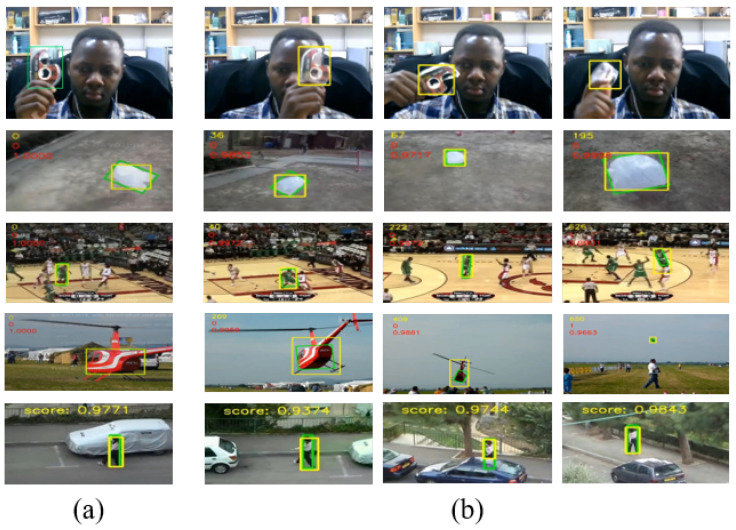
Illustration of the visual object tracking results: (**a**) The initial frame with a bounding box. (**b**) Tracking results in three different selected frames with the ground truth in green and ours in yellow bounding boxes. From top to bottom: Scotch tape webcam, Bag, Basketball, Helicopter and Woman, respectively. Apart from Scotch tape webcam, videos are from VOT2018 [9] and OTB2015 [10] datasets.

The most challenging part of visual tracking is the real-time or online tracking as shown in Figure 1, where the tracker cannot use future frames to infer the current position of an object [8].

In this work, we address the accuracy gap and frame update problems of the Siamese network and correlation filters, respectively, in a twofold contribution:

- We extract spatially semantic correlation features (SSF) from the Siamese network.
- We learn adaptive correlation filters (ACF) at every convolutional layer output and calculate their weighted sum in the end.

In the reminder of this paper, we briefly review related works in Section 2, followed by the proposed method in Section 3. In Section 4 and Section 5, we implement and evaluate our method. Finally, we conclude the paper in Section 6.

## 2. Related Works

In this section, we briefly describe deep Siamese tracking and correlation filters in Section 2.1 and Section 2.2, respectively.

### 2.1. Deep Siamese Tracking

Zhang et al. [1] and Li et al. [2] have recently proved that the Siamese network can benefit from deeper backbone networks using end-to-end learning. Based on those works, Siamese networks formulate object tracking as a cross-correlation problem between two input signals, one of which is an interested region of an image, and the other is a relatively larger search window in another image [1,2,5,6]. Training the Siamese network involves a Y–shaped network that joins two branches, one of which predicts the object template (interested image), and the other predicts the search region (search window). This process consists of two steps—(1) an offline training [1,5] for a similarity function learning between the two input signals by cross-correlating them, and (2) an online training for the similarity function update as the tracking goes on [5]. With the addition of spatially semantic correlation features (SSF) and adaptive correlation filters (ACF), we improved both accuracy and speed of the deep Siamese networks.

### 2.2. Correlation Filter Tracking

Correlation filters have attracted attention in the tracking field during the last decade due to their high computational efficiency in the Fourier domain and the kernel trick method [11,12]. This consists of a form of circular shifts of input signals to a target Gaussian function which does not require hand-crafted features of the target. Correlation filter related works, HOG or color-attributes presented a frame update problem and used hand-crafted features [12]. Therefore, we address these by finding multiple correlation filters in hierarchical convolutional layers as opposed to only one single filter at the classification/regression layer of the network used by existing approaches.

## 3. Proposed Method

**Figure 2:**
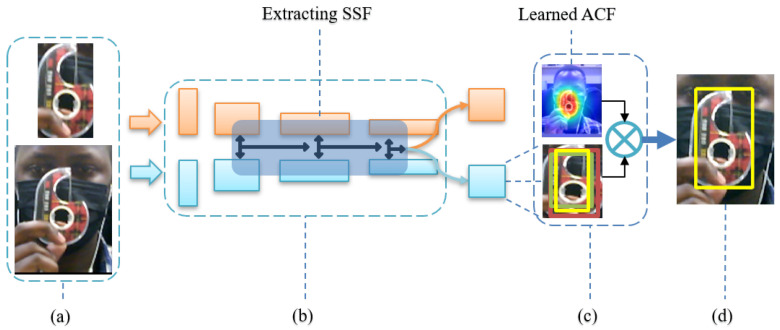
Spatially semantic correlation features (SNS-CF) proposed algorithm: (**a**) A pair of input images and the corresponding search window, (**b**) the Siamese network with three region proposal networks in a shadowed middle part, which highlights spatially semantic correlation features of interest, (**c**) the correlation search module, with multiple up-sampled bounding boxes convolved with a learned adaptive correlation filter (ACF), and (**d**) output of the predicted final tracking result with a yellow bounding box.

This section describes the proposed algorithm as shown in Figure 2 and revisits the twofold contributions, as mentioned in the previous section. It will as well explain preliminaries to understand the proposed contributions.

### 3.1. Siamese Net

Bertinetto et al. first proposed the Siamese network, called SiamFC [5], and Li et al. improved it by using region proposal networks [6]. Recently, Li et al. made further improvements by solving the problem of a small receptive field, network stride and padding while reducing the translated image z and a candidate search image x. The image z represents the object of interest, while x is typically larger and represents the search area in subsequent video frames. Both inputs are processed by a ConvNet φ with parameters θ. This yields two feature maps, which undergo a cross-correlation:

(1) $$f_{\theta }\left(z,x\right)=\varphi_{\theta }\left(z\right)*\varphi_{\theta }\left(x\right)+b\_1,$$

where b_1 denotes a positive offset to model the similarity value. This ensures the efficient training and inference by obeying intrinsic restrictions for structure symmetry, that is, $f\left(z,x^{\prime }\right)=f\left(x^{\prime },z\right)$, which is appropriate for the similarity learning. Equation (1) performs an exhaustive search of the pattern *z* over the image *x* to match the maximum value in the response map *f* to the target location. This is done through an offline training with random image pairs x,z taken from training videos and the corresponding ground truth label *y*. The parameters θ of the ConvNet φ are obtained by minimizing the logistic loss L over the training set:

(2) $$\underset{\theta }{\arg\min}\underset{\left(z,x,y\right)}{E}L\left(y,f_{\theta }\left(z,x\right)\right).$$

### 3.2. Region Proposal Network (RPN)

The Siamese network weights the similarity measure between the input image and the search window. We need an extra fragment installed in adjacent layers of the network, and the choice of where and how many is a hyperparameter. This extra fragment is used to refine the proposal. It consists of a pair-wise correlation section with two branches as well, one for classification of background and/or foreground, and another for regression of proposal. More about these RPNs are found in a pioneering work by Li et al. [6]. We made three RPNs and implanted them in our modified ResNet50 [13] to capture spatially semantic information. RPN1, RPN2 and RPN3 aggregate multi-branch features of conv 3 (res3d_branch2c), conv4 (res4f_branch2c) and conv5 (res5c_branch2c), respectively. The extraction of such information used in tracking tasks follows in the next section.

### 3.3. Extracting SSF

**Figure 3:**
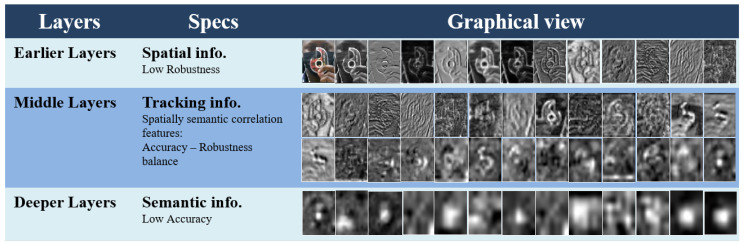
Graphical view of spatially semantic correlation features. From Left to right, top to bottom we have a sequence of activations from a deep convolutional neural network (CNN) (ResNet50 [13] for our case), where the first (top) row consists of Earlier layer activations poor in robustness, and the last (bottom) row consists of deeper layer activations poor in accuracy. To balance the robustness—accuracy trade-off, our method suggests to use middle layer activations (2 middle rows) rich in both spatial and semantic information.

We aggregate different deep layers into RPNs following Reference [6]. The three RPNs are located on the richest middle layers, as shown in Figure 3.

The idea of extracting SSF comes from the need to improve existing features. Dimitris et al. significantly improved classification features by applying robust optimization techniques [4]. On the other hand, Erhan et al. decided on good features to correlate for visual tracking by utilizing robust features that are invariant to any kind of appearance change of the object, while predicting the object location as properly as in the case of no appearance change [14]. Other approaches used hierarchical features [3], spatially semantic features [7] and hierarchical attention weights [15] to define appropriate features for object tracking in CNNs.

In our task, we use aggregated layers in RPNs to collaboratively infer the target localization. As for ResNet50 [13], we explore multi-level features extracted from the last three aggregated layers. We refer to these outputs RPN1, RPN2 and RPN3 as $x_{3}$, $x_{4}$ and $x_{5}$, respectively. They constitute both scores S and bounding boxes B and as we mentioned before, we drop scores and use only bounding boxes. We will perform an interpolation of B as shown in Figure 2, to have the same spatial resolution, (see Section 3.6) to be able to perform a correlation search. At each RPN we perform a weighted sum directly as they have same individual spatial resolution, and a weighted-fusion layer combines all the outputs as:

(3) $$B_{all}=\sum_{l=3}^{5}\beta_{i}*B_{l},$$

where $B_{all}$ denotes the bounding boxes on weighted-fusion layer, $B_{l}$ denotes the bounding boxes on *l*th layer and $\beta_{i}$ denotes the interpolation factor.

### 3.4. Convolutional Features

Extraction of convolutional feature maps encodes target appearances. The forward propagation along the network strengthens semantic discrimination, while the spatial information gradually reduces. As shown in Figure 1, it is easy to locate the scotch tape in earlier layer activation maps, but it gets blurry in deeper network layers. Since only middle layers conserve spatially semantic information, we ignore both earlier and deeper layers, and put our focus on middle layers [7,15]. Conventionally, CNNs use different operators, pooling being one of them, which result in shrinking the spatial resolution with the increase in the depth of convolutional layers. For instance, the size of res5a_branch2b, the 145th convolution of ResNet50 [13] is 7×7×512 which is $\frac{1}{32}$ of the input size of 224×224×3. To preserve the spatial resolution, we bilinearly interpolate each feature map to a fixed size as:

(4) $$x_{i}=\sum_{k}\alpha_{ik}h_{k},$$

where $\alpha_{ik}$ denotes the interpolation weight and i,k denote the position of neighboring feature vectors, respectively. More details on connecting features from multiple layers are found in Reference [16] for segmentation and fine-grained localization using CNNs.

### 3.5. Correlation Filters

Typical correlation filters [7,17,18] learn a discriminative classifier and estimate the translation of target objects by searching for the maximum correlation response. Correlation filters have been very competitive, thanks to working in the Fourier domain, where circular shifts are computed in a lapse of time using kernel trick [11,12]. The circular shifts are defined as:

(5) $$x=\left\{x_{m,n}|\left(m,n\right)\in \left\{0,1,\ldots ,M-1\right\}\times \left\{0,1,\ldots ,N-1\right\}\right\},$$

where *x* denotes the *l*th layer of feature vector of size M×N×D. M,N and *D* denote width, height, and number of channels, respectively. $x^{l}$ concisely denotes *x* on the layer *l*, implicitly with its dependencies, M,N and *D*. M−1 and N−1 denote the circular forms of *x* in both directions. The common characteristic of circular shifts is their Gaussian function label y(m,n), determined as:

(6) $$y\left(m,n\right)=e^{-\frac{{\left(m-M/2\right)}^{2}+{\left(n-N/2\right)}^{2}}{2\sigma^{2}}},$$

where σ denotes the kernel width. A correlation filter *w* with the same size of *x* is then learned by solving the following minimization problem:

(7) $$w^{*}=\underset{w}{\arg\min}\sum_{\left(m,n\right)}||w.x_{m,n}-{y||}^{2}+{\lambda ||w||}_{2}^{2},$$

where λ denotes a positive regularization parameter, and the inner product is induced by a linear kernel in Hilbert space [3]. The core ingredients in CNNs are the ability to learn by training and avoiding handcrafted samples. Therefore, the correlation filter in the Fourier domain described in (7) can save a tremendous amount of time by solving it in each individual feature channel using the fast Fourier transform (FFT). Capital letters denote the corresponding small letter signals in Fourier transformed signals. The learned filter in the frequency domain on the *d*th (*d*∈{1,…,D}) channel can be written as:

(8) $$W^{d}=\frac{Y⊙{\bar{X}}^{d}}{\sum_{i=1}^{D}X^{i}⊙{\bar{X}}^{i}+\lambda },$$

where *Y* is the Fourier transform of $y=\left\{y_{m,n}|\left(m,n\right)\in \left\{0,1,\ldots ,M-1\right\}\times \left\{0,1,\ldots ,N-1\right\}\right\}$, following (5), and ⊙ denotes the Hadamard (element-wise multiplication) product operator.

### 3.6. Learned ACF

Also known as the maximum of the correlation response map, given an image patch in the next frame, the feature vector on the *l*th layer is denoted as *z* of size M×N×D. The *l*th correlation response map is computed as:

(9) $$f_{l}=F^{-1}\left(\sum_{d=1}^{D}W^{d}⊙{\bar{z}}^{d}\right),$$

where $F^{-1}$ denotes the inverse FFT operation. The learning of ACF is completed in searching for the position of the maximum value of Equation (9) with with the same size. It is cross-correlated with interpolated bounding boxes (B) in Section 3.3, to find the optimized target location.

## 4. Implementation Details

SNS-CF algorithm is a modified ResNet50 [13] to perform proposal classification and bounding box regression. We added three 1×1 randomly initialized convolutional layers to conv3, conv4, and conv5 to reduce the feature dimension down to 256. During training, it is optimized using Stochastic Gradient Descent (SGD) method, which can benefit from parallel computing using 8 GPUs with a total of 128 pairs per minibatch, that is, 16 pairs per GPU, to reduce a week of training into just 12 h. We initially used a single GPU with 16 pairs, initial learning rate of 0.001 for first 5 epochs to train RPN branches. The entire network is trained in an end-to-end manner, and in the end, 15 last epochs are trained with an exponential learning rate decay from 0.004 to 0.0004, with a momentum of 0.9. The training loss is the sum of standard smooth loss L in (2) and the correlation filter loss $w^{*}$ in (7).

## 5. Experimental Results

Hardware specifications—SNS-CF algorithm is implemented using Python [19] and evaluated on MATLAB (Natick, MA, USA) [20] LaSOT evaluation toolkit [21], Intel [22] i7-8700K 3.70 GHz CPU with 32 Mb RAM and a single NVIDIA (Santa Clara, CA, USA) [23] GeForce GTX 1080 Ti. Dataset—SNS-CF algorithm is evaluated on widely used tracking datasets, for instance OTB-2015 [10], VOT-2018 [9], and LaSOT [21]. OTB-2015 [10] consists of 100 video sequences, VOT-2018 public dataset [9], one of the most recent datasets for evaluating online model-free single object trackers consists of 60 video sequences while LaSOT [21] dataset provides a large-scale, high-quality dense annotations with 1400 videos in total and 280 videos in the training set.

Metrics—OTB-2015 [10] is evaluated following the evaluation protocol in Reference [10], and has three following metrics, Distance Precision rate (DP), Overlap ratio (OS), and Center Location Errors (CLE). VOT-2018 [9] is evaluated following the evaluation protocol in Reference [9]. We adopt the Expected Average Overlap (EAO), Accuracy (A), Robustness (R), and no-reset-based Average Overlap (AO) to compare different trackers. Lastly, LaSOT [21] is evaluated following evaluation protocol in Reference [21] with Distance Precision (DP) and Overlap Success (OS) plots over 100 benchmark sequences using One-pass evaluation (OPE) on both threshold and Area Under the Curve (AUC). We will present the evaluation results with respect to each dataset shortly.

Training—The backbone network of SNS-CF algorithm is ResNet50 [13] pre-trained on ImageNet [24] for image labeling, as a good initialization to other tasks, even though it is quite old now. In both training and testing, we followed SiamFC [5] protocol and used an exemplar and search images patches of 127×127 and 255×255 pixels respectively. We randomly translated up to ±8 pixels and re-scaled $2^{\pm 1/8}$ and $2^{\pm 1/4}$ for exemplar and search images, respectively. We trained our network on the training sets of Imagenet-VID [24], COCO [25], and Youtube-VOS [26].

Evaluation method—We perform the evaluation of our algorithm with respect to correlation filter—based trackers, and Siamese network—based trackers. We will conduct separate evaluation and provide results for each category. Starting from correlation filter—based trackers, we quantitatively evaluated the proposed algorithm with 9 state-of-the-art trackers [3,12,27,28,29,30,31,32,33], considering the distance precision rate (DP) at 20 pixels, overlap success rate (OS) at 0.5, center location errors (CLE) and tracking speed, from 100 sequences of OTB-2015 [10] benchmark.

Second, the proposed algorithm is evaluated compared to Siamese networks—based trackers, and we will focus on the short-term single object racking on OTB2015 [10] and VOT2018 [9], and analyze the generalization of our method on LaSOT [21], the most recent largest benchmark for single object tracking. Short-time single object tracking, as opposed to long-term single object tracking is the scenario where the object has to stay in the field of view throughout the tracking, or just for a fraction of time leaves the field of view or becomes fully occluded.

CF–based results—We present results from evaluating the proposed algorithm with respect to 8 correlation filter—based state-of-the-art trackers [3,12,27,28,29,30,31,32,33]. They can be broadly categorized into three classes that is, deep learning trackers (DL–Trackers) [27], correlation filter trackers (CF–Trackers) [12,28,34] and representative online classifier trackers (ROC–Trackers) [29,31,32,33]. Table 1 illustrates the quantitative comparisons of distance precision rate (DP) at 20 pixels, overlap success rate (OS) at 0.5, center location errors (CLE), and tracking speed, from 100 sequences of OTB-2015 [10] benchmark. It shows a favorable performance against the state-of-the-arts.

Table 1 shows the highest tracking result against state-of-the-art trackers in terms of DP, OS and CLE, which are roughly comparable to Reference [3]. KCF [12], second fastest and STC [28], the fastest use handcrafted features, which do not require high computational complexity sand time as deep CNN feature—based do. However, our tracker runs at an average of 35.1 fps, which is fairy good among CNN—based trackers.

Siamese–based results—We present results from evaluating the proposed algorithm with respect to VOT-2018 [9] and LaSOT [21] benchmarks. First, we start from VOT-2018 [9] and test our tracker SNS-CF against 7 state-of-the-art methods containing either correlation filters or Siamese networks or both [2,6,35,36,37,38,39,40,41,42]. We follow its evaluation protocol and present results in the following Table 2.

Takeaways from Table 2 are interesting as we can notice that the proposed algorithm achieves the best Expected Average Overlap rate (EAO) against all the state-of-the-arts, with a gain of roughly 1% to the baseline and top performing. The accuracy is about 1.3% short of the baseline, but also higher than any other state-of-the-art. The robustness is 1.1% higher than the baseline, but unfortunately still lower than the VOT-2018 [9] challenge winner MFT [39], mostly because the latter is armed with Multi-hierarchical independent correlation filters, a close technology to our algorithm. Notice that we outperform it in the rest of the metrics. Lastly, the overall One Pass Evaluation (OPE) is also adopted to evaluate trackers and the AO values are reported to demonstrate their performance. Our algorithm achieved second best value to the beseline and overall benchmark.

**Figure 4:**
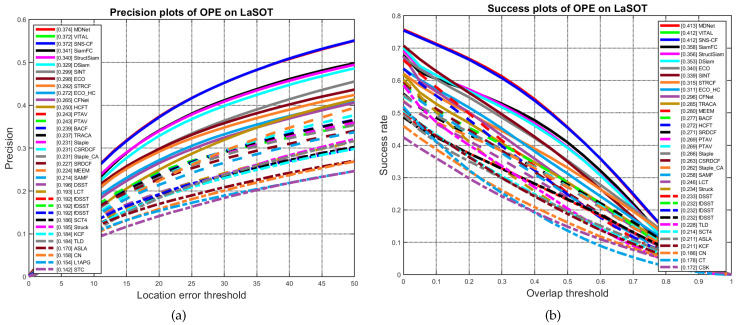
(**a**) Distance Precision and Overlap Success plots over 100 benchmark sequences using One-pass Evaluation (OPE) on both threshold scores at 20 pixels and (**b**) Area Under The Curve score (AUC) (right) on LaSOT [21]. Notice that the proposed algorithm (Blue) ranks third, with first three trackers MDNet [43], VITAL [44] and SNS-CF (ours) merely having the same performance. Extensive assessments over fifteen challenging tracking scenarios are experimented and results are available from authors upon request. We reproduced Figure 4 using MATLAB official LaSOT Evaluation toolkit [21].

Second, we further validate the proposed algorithm by testing it on a larger and more challenging dataset, LaSOT [21]. We follow its evaluation protocol and report the overall performances in Figure 4.

Fusion–based results—We present results from combining state-of-the-arts of both correlation filter–based tracker [3] and Siamese network–based tracker [2] with direct combination, that is, with no modification, and with our proposed algorithm that includes the extraction of SSF and the learning of ACF. The following Table 3 has the details.

Table 3 on the preceding page shows that SNS-CF algorithm clearly improves both the correlation filter and Siamese network trackers in a number of metrics. The last column indicates the direct combination of correlation filter tracker [3] and Siamese network [2] without our contributions, and we remark an early improvement in CLE, EAO, robustness and speed, thanks to both the advantages of deep CNN features as opposed to handcrafted HOG features, and the Fourier domain of correlation filters that dramatically improves the speed [11,12]. The first column shows that the proposed algorithm outperforms both correlation filter and Siamese network baselines in general, thanks to spatially semantic (SSF) features and the learning of adaptive correlation features (ACF).

Table 4 shows that SNS-CF performs best on both intra-class and illumination variations, while it is the second best on occlusions due to the lack of a re-detection module. On the whole, the proposed SNS-CF shows a significant improvement in robustness.

**Figure 5:**
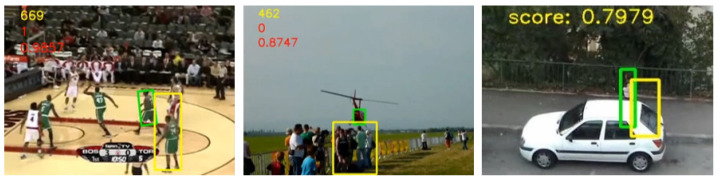
Failure cases. Video instances of Basketball, Helicopter and Woman on VOT-2018 [9] and OTB2015 [10]. They represent multiple foreground images similar to the target, severe out-of-view and sudden background clutter respectively.

Failure cases—In some challenging scenarios, our algorithm failed completely to locate to position of the target. We suspect this is due to intense background clutter, appearance of many similar foreground images, although not targets, and severe out-of-view. Some other cases include bright background and dark foreground, where the first layer features are enough to check failure instead of using all the SSF features. Severe out-of-view cases may be well addressed if our algorithm was equipped with a re-detection module, which will be our future research. This is illustrated in Figure 5, whereas correctly located targets are illustrated in Figure 1.

## 6. Conclusions

In this paper, we proposed a novel effective fusion algorithm called SNS-CF, which trains a Siamese network and a correlation filter for visual object tracking. We used the fading correlation filter technology to improve the popular Siamese network. The similarity search technique of a typical Siamese network, fused with correlation filter, alongside spatially semantic correlation features from hierarchical layers produces a fast, robust and accurate SNS-CF algorithm for visual object tracking. We believe this is going to open a room for improvement about such a fusion. Extensive experimental results on large datasets include LaSOT [21], VOT-2018 [9] and OTB-2015 [10], and shows the effectiveness of SNS-CF algorithm by achieving state-of-the-art results.

## Figures and Tables

**Table 1:** Quantitative evaluation of SNS-CF algorithm with eight state-of-the-art trackers [3,12,27,28,29,30,31,32,33], considering the distance precision rate (DP) at 20 pixels, overlap success rate (OS) at 0.5, center location errors (CLE) and tracking speed, from 100 sequences of OTB-2015 [10] benchmark. Red and blue numbers indicate the best and second best results, respectively.

Metrics	Ours (SNS-CF)	CF2 [3]	KCF [12]	Struck [29]	DLT [27]	STC [28]	TLD [33]	MIL [32]	CT [31]
DP rate ↑ (%)	84.0	83.7	69.2	63.5	52.6	50.7	59.2	43.9	35.9
OS rate ↑	65.7	65.5	54.8	51.6	43.0	31.4	49.7	33.1	27.8
CLE ↓ (pixel)	20.2	22.8	45.0	47.1	66.5	86.2	60.0	72.1	80.1
Speed ↑ (FPS)	35.1	10.4	243	9.84	8.43	653	23.3	28.0	44.4

**Table 2:** Comparison with the state-of-the-art trackers in terms of Expected Average Overlap (EAO), Robustness and Accuracy on the VOT-2018 [9]. Red and blue numbers indicate the best and second best results, respectively.

Metrics	Ours (SNS-CF)	SiamRPN++ [2]	LADCF [38]	MFT [39]	SiamRPN [6]	UPDT [40]	SA_Siam_R [41]	DRT [42]
EAO ↑	0.423	0.414	0.389	0.385	0.383	0.378	0.337	0.356
Accuracy ↑	0.587	0.600	0.503	0.505	0.586	0.536	0.566	0.519
Robustness ↓	0.223	0.234	0.159	0.140	0.276	0.184	0.258	0.201
AO ↑	0.487	0.498	0.421	0.393	0.472	0.454	0.429	0.426

**Table 3:** Comparison with correlation filter, Siamese network, and the proposed SNS-FC algorithm that fuses both technologies. We present the results on VOT-2018 [9]. Red and blue numbers indicate the best and second best results, respectively.

Metrics	Ours (SNS-CF)	Correlation Filter [3] (Baseline)	Siamese Network [2] (Baseline)	Both (Without SSF and ACF)
DP rate ↑ (%)	84.0	83.7	-	83.3
OS rate ↑ (%)	65.7	65.5	-	65.0
CLE ↓ (pixel)	20.2	22.8	-	21.3
EAO ↑	0.423	-	0.414	0.420
Accuracy ↑	0.587	-	6.00	0.557
Robustness ↓	0.223	-	0.234	0.228
Speed ↑ (FPS)	35.1	10.4	-	34.2
AO ↑	0.487	-	0.489	0.393

**Table 4:** Comparison of robustness on different SOT challenging problems with correlation filter, Siamese network, and the proposed SNS-FC algorithm. We present the results on VOT-2018 [9]. Red and blue numbers indicate the best and second best results, respectively.

Scenarios	Ours (SNS-CF)	Correlation Filter [3] (Baseline)	Siamese Network [2] (Baseline)
Background clutter	0.228	0.225	0.230
Intra-class variations	0.198	0.302	0.227
Occlusions	0.312	0.226	0.232
Illumination variations	0.154	0.159	0.247
Average Robustness ↓	0.223	0.228	0.234
